# Supplementary material for: Outcomes and Predictors of In-Hospital Mortality among Older Patients with Dementia
Source: J Clin Med. 2022 Dec 21;12(1):59. doi: 10.3390/jcm12010059 (PMC9821230; doi:10.3390/jcm12010059)

Description of propensity score match

In order to evaluate the impact of dementia on the in-hospital outcome of the affected patients, we compared patients affected by dementia with a control group with similar clinical characteristics, and similar acute conditions. The control group was selected by a propensity score matching (PSM) analysis among all the patients admitted in the same period not affected by dementia. PSM was generated using a logistic regression model on the baseline covariates considered as potentially influencing the in-hospital outcome, and adding the main clinical diagnosis and the symptoms at admission.

The variables considered for PSM were:

- Age
- Gender
- Charlson Comorbidity Index
- NEWS score at admission
- Primary ED diagnosis (based on ICD-10 Codes)
- Symptoms at ED admission (acute neurological symptoms, respiratory distress, trauma, fever, chest pain, abdominal pain, syncope, diarrhea, vomit, constipation, and malaise/fatigue)

Patients were then matched on these propensity scores with a ratio 1:1.

An optimal matching with a caliper size of 0.2 was used to avoid poor matches.

A description of PSM analysis and distribution before and after match is provided in supplementary Figure 1 and 2.

Data were analyzed using SPSS for Windows, version 25 (SPSS Inc., Chicago, IL, USA).

Table S1. Sample sizes before and after PSM

All cases		Matched		Unmatched	
**Control**	**Treated**	**Control**	**Treated**	**Control**	**Treated**
45402	3560	3559	3559	41843	1

Figure S1. R graph of study population before and after propensity score matching.

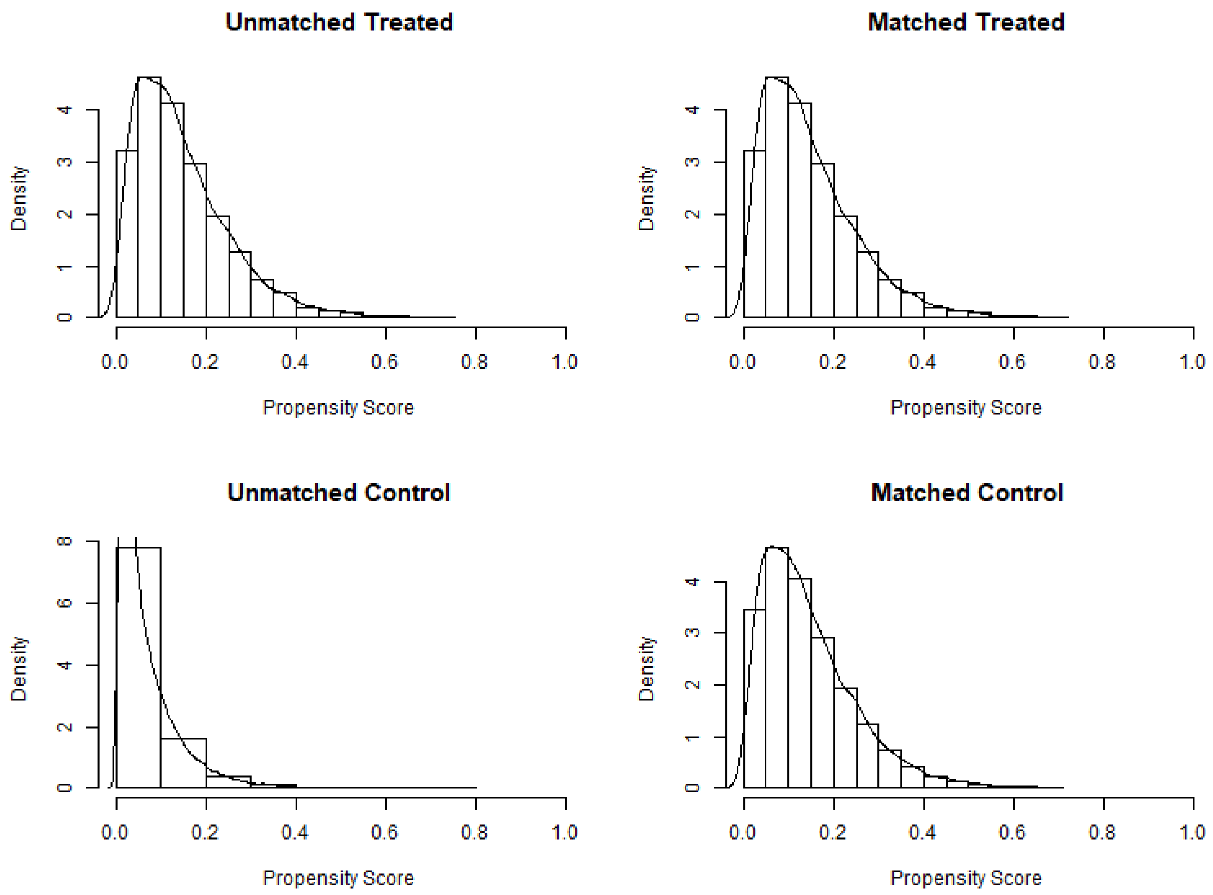

Figure S2. Distribution of the standardized differences before and after the matching. No patients was discarded in the analysis. Overall balance (Hansen & Bowers) chi^2 was 14.840; p=0.607. Relative multivariate imbalance L1 (Iacus, King, & Porro) was 0.890 before matching and 0.861 after matching. No covariates showed a large imbalance (|d|>0.25)

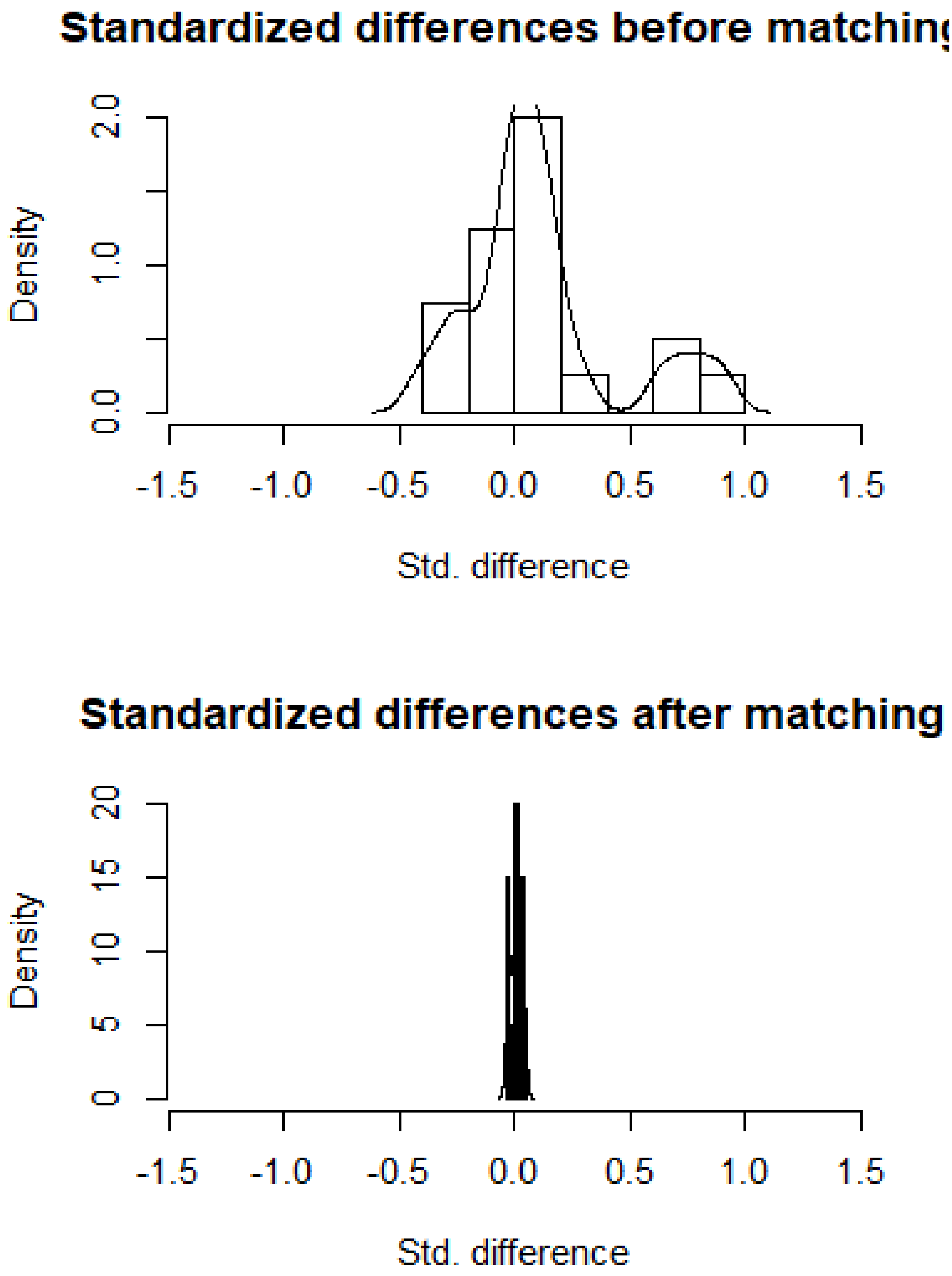

Supplement: Supplementary file 1 [file jcm-12-00059-s001.zip › jcm-1991634-supplementary.pdf]
